# Preventable Hospitalizations for Hypertension: Establishing a Baseline for Monitoring Racial Differences in Rates

**DOI:** 10.5888/pcd10.120165

**Published:** 2013-02-14

**Authors:** Julie C. Will, Paula W. Yoon

**Affiliations:** Author Affiliation: Paula W. Yoon, Centers for Disease Control and Prevention, Atlanta, Georgia.

## Abstract

**Introduction:**

Preventable hospitalization for hypertension is an ambulatory care–sensitive condition believed to indicate the failure of outpatient and public health systems to prevent and control hypertension. Blacks have higher rates of such hospitalizations than whites. The 2010 Patient Protection and Affordable Care Act (PPACA) seeks to implement higher quality health care, which may help close the racial gap in these rates. The objective of this study was to analyze pre-PPACA baseline rates of preventable hypertension hospitalizations in the United States and racial differences over time.

**Methods:**

We used data from the 1995–2010 National Hospital Discharge Survey, a stratified, probability-designed survey representing approximately 1% of hospitalizations in the United States. Rates were calculated using specifications published by the Agency for Healthcare Research and Quality requiring census data as denominators for the rates. We combined at least 3 years of data to obtain more precise estimates and conducted a trend analysis by using rates calculated for each of the resulting 5 periods.

**Results:**

For both sexes, all age groups, and each period, blacks had higher crude rates than whites. Age- and sex-standardized rates confirmed this finding (eg, 2007–2010: blacks, 334 per 100,000; whites, 97.4 per 100,000). Rates were generally flat over time; however, white women aged 65 or older showed increasing rates.

**Conclusion:**

Using national data, we confirmed higher rates of preventable hypertension hospitalizations for blacks, showing little improvement in disparities over time. This pre-PPACA baseline for blacks and whites allows for ongoing monitoring of preventable hospitalizations for hypertension.

## Introduction

Improving access to good-quality health care, controlling health care costs, and promoting prevention are important policy issues in the United States (1). Many factors influence access to and receipt of good-quality health care (2). In 2009, more than one-fifth (40 million) of the US population aged 18 to 64 years lacked health insurance (3). During the early 2000s, only 55% of adults living in 12 metropolitan areas of the United States received care according to established national guidelines (4). Multifaceted strategies are required to address the many factors associated with lack of access to and quality of health care (4).

Experts have identified conditions for which hospitalization could be prevented if patients receive early access to good-quality health care and have labeled them ambulatory care–sensitive conditions (ACSCs) (5,6). Hospitalization for hypertension is an ACSC that is believed to indicate the failure of the public health and outpatient health care systems to prevent and control high blood pressure in communities (7).

In 1993, the Institute of Medicine (IOM) issued a report on access to health care in the United States and the unacceptable existence of racial disparities in such access (8). Using ACSCs and 1988 data from hospitals in 11 states, the IOM showed that low-income populations were almost 8 times as likely to be hospitalized as high-income populations. That same year, for hypertension hospitalizations, race was an even more powerful predictor than was income (5).

The 2010 Patient Protection and Affordable Care Act (PPACA) aims to make primary care and preventive services more accessible, affordable, and effective (9). Tracking changes in ACSCs may be a way to monitor the effect of health care reform on population health.

Higher rates among blacks than whites have been found at the state level (10,11) and across multiple states (12,13). One study examined state-specific rates over time (10); however, the data used were more than a decade old. The objective of this study was to analyze pre-PPACA baseline rates of preventable hypertension hospitalizations in the United States and racial differences over time. We used recent data on all adults (not only those enrolled in Medicare) from a nationally representative hospitalization database. 

## Methods

### Data source and definitions

We used data from the 1995–2010 National Hospital Discharge Survey (NHDS) conducted by the National Center for Health Statistics (NCHS) (14) to analyze temporal trends by race. NHDS is a stratified, probability-designed survey with 3 stages of sampling. At the first and second stages of sampling, the NHDS selects noninstitutional hospitals in geographic areas of the United States, exclusive of federal and Veterans Administration hospitals. At the third stage of sampling, the NHDS selects a systematic random sample of inpatient discharge records from each participating hospital, representing approximately 1% of all hospitalizations in the United States. Data collection for NHDS was approved by the NCHS Research Ethics Review Board. Analysis of de-identified data from the survey is exempt from the federal regulations for the protection of human research participants.

Only short-stay hospitals (average length of stay <30 days) and general hospitals (medical or surgical) with at least 6 beds for inpatient use are included in the NHDS. The survey uses a sample of hospitals drawn in 1987, updating them every 3 years to account for hospitals that have opened, closed, or changed their status. From 1995–2007, the sample included between 501and 525 hospitals (unweighted response rate, 89%). From 2008–2010, the NHDS used a half-sample of 239 hospitals (unweighted response rate, 86%).

We calculated population-based hospital discharge rates by using the specifications published by the Agency for Healthcare Research and Quality (AHRQ) for prevention quality indicator number 7, hypertension admission rate (15). The AHRQ specifications have been endorsed by the National Quality Forum in 2007 (16) and have been adopted by the Organisation for Economic Cooperation and Development for making cross-country health comparisons (17). We calculated the numerator using all nonmaternal discharges of people aged 18 years or older with the *International Classification of Diseases, 9th Revision, Clinical Modification* (ICD-9-CM) principal diagnosis codes for hypertension, which include any of the following: 401.0, 401.9, 402.00, 402.10, 402.90, 403.00, 403.10, 403.90, 404.00, 404.10, and 404.90. The specifications indicate that maternal discharges are to be identified using major diagnostic category 14 (pregnancy, childbirth, and puerperium) and then excluded from the numerator. Until recently, NHDS data were not grouped by major diagnostic codes; thus, maternal discharges were identified according to a method that uses ICD-9-CM codes alone to, as closely as possible, replicate major diagnostic category 14 (18). As specified in the AHRQ instructions, we excluded from the numerator transfers from another institution (database missing this information before 2001) and discharges with cardiac procedure codes in any field. Finally, we eliminated discharges with any diagnosis of stage I through IV kidney disease, if accompanied by procedures for preparation for hemodialysis. We calculated denominators for rates by using US census population estimates published by the NCHS as part of the documentation package for each year’s NHDS database (14).

Demographic and medical information were abstracted from the face sheets of hospital records using a medical abstract form by staff working for NCHS or by abstraction service staff. After editing and quality checks were completed, the data were made available to researchers through a computerized database. We used the confidential database that included variables restricted from public use. This allowed us to use sampling design variables to calculate standard errors for estimates. We used racial category as the descriptor of primary interest. We used 2 categories of race: white and black. Both categories include Hispanics; only 25% of the abstract forms specified Hispanic or non-Hispanic ethnicity (19). We also created an “other” category that consisted of Asian, American Indian, Alaska Native, Eskimo, Native Hawaiian, Pacific Islander, and any other race specified on the form. Race was considered missing if the box specifying “not stated” or multiple boxes specifying race were checked. We included the multiracial category in the missing category because it has been coded in the database only since 2000 and accounted for a small percentage of hospitalizations (eg, during 2004–2006 it was 0.16%). Although we used the “other” category in a description of hospitalizations for hypertension, we did not use it for the main analysis because the sample sizes were small and had broad confidence intervals. We used the 4 geographic regions of the US Census Bureau: Northeast, Midwest, South, and West (20). For insurance type, we used 4 categories; Medicare, Medicaid, private insurance, and all other. The patient’s report of the expected principal source of payment was used to derive these categories. “All other” included types such as other government insurance, self-pay, hospitalization without a charge, and worker’s compensation.

### Statistical analysis

We estimated the total weighted number of hospitalizations for hypertension each year from 1995–2010 for people aged 18 years or older. Because hypertension as a primary reason for hospitalization is rare and because we studied minority populations, we combined at least 3 years of data to obtain more precise estimates. Consequently, we analyzed 5 periods: 1995–1997, 1998–2000, 2001–2003, 2004–2006, and 2007–2010. Because of the use of half-samples in 2008, 2009, and 2010, we combined 4 years of data for the last period to obtain more precise estimates. We summed population estimates over these same periods and calculated rates per 100,000 population. Because we wanted to describe the burden of hospitalizations in various population subgroups, we report crude rates stratified by age category (18–44, 45–64, and ≥65), sex, and race (white or black). We standardized rates by period, age, and sex to the 2000 US Census population.

We used SUDAAN version 10.0 (Research Triangle Institute, Research Triangle Park, North Carolina) to calculate standard errors, variances, and 95% confidence intervals for the estimates of the number of hospitalizations for hypertension (the numerator) during each 3- or 4-year period. We did not calculate confidence intervals for the denominators because we derived the denominators from a census of the population. We calculated 95% confidence intervals for the rates by dividing the variance of the numerator by the denominator squared and then taking the square root of the result multiplied by 1.96. We used simple linear regression, weighted by the inverse of the variance (from SUDAAN) of each period’s weight, to test for time trends in both the crude and standardized rates for each subgroup of interest. We used *z* tests to calculate differences in rates between groups. Significance was set at *P* < .05.

## Results

During 1995–2010, 35,503 preventable hospitalizations for hypertension occurred among people aged 18 or older in the NHDS-sampled hospitals, which translated to a weighted number of 4,758,728 US hospitalizations. Most hospitalizations occurred among people aged 45 or older; only small differences were found in the percentages between those aged 45 to 64 years and those aged 65 or older (Table 1). Approximately 60% of the hospitalizations occurred among women, and hospitalizations occurred more frequently in the South than in other regions. The most frequent payer for a hypertension hospitalization was Medicare (range, 43%-45%), and private insurance was the second largest payer (range, 31%-39%). In 1995–1997, Medicaid paid for almost 8% of the hospitalizations for hypertension; however, in 2007–2010, this number had risen to more than 12%.

**Table 1:** Preventable Hospitalizations for Hypertension, by Selected Demographic Characteristics, National Hospital Discharge Survey, United States, 1995–2010

Characteristic	Period, % (95% Confidence Interval)				

	1995–1997 (n = 657,190)	1998–2000 (n = 770,799)	2001–2003 (n = 928,684)	2004–2006 (n = 940,826)	2007–2010 (n = 1,461,229)
**Age, y**					
18–44	16.0 (14.5–17.6)	15.7 (14.2–17.4)	13.7 (12.5–15.2)	14.1 (12.8–15.4)	13.2 (11.7–14.8)
45–64	39.1 (36.7–41.6)	40.8 (38.8–42.9)	40.3 (38.2–42.5)	42.7 (40.6–44.7)	41.6 (39.3–43.9)
≥65	44.9 (42.1–47.7)	43.4 (41.3–45.6)	46.0 (43.6–48.4)	43.3 (41.2–45.4)	45.2 (42.8–47.6)
**Sex**					
Male	39.3 (37.1–41.6)	38.1 (35.8–40.5)	39.2 (37.3–41.2)	38.5 (36.4–40.6)	41.2 (39.1–43.4)
Female	60.7 (58.4–62.9)	61.9 (59.5–64.2)	60.8 (58.8–62.7)	61.5 (59.4–63.7)	58.8 (56.6–61.0)
**Race^a^**					
White	68.8 (65.5–72.0)	65.5 (61.6–69.2)	67.7 (63.7–71.5)	66.9 (62.9–70.8)	65.4 (60.6–69.8)
Black	26.9 (24.0–30.1)	28.8 (25.5–32.4)	27.9 (24.3–31.8)	27.1 (23.6–30.9)	29.1 (25.1–33.4)
Other	4.3 (3.1–5.8)	5.7 (4.3–7.6)	4.5 (3.4–5.8)	6.0 (4.7–7.7)	5.5 (4.2–7.2)
**Region**					
Northeast	22.7 (20.1–25.5)	23.9 (20.5–27.7)	22.4 (19.5–25.6)	23.8 (20.9–27.0)	23.6 (18.9–29.1)
Midwest	20.1 (17.5–22.9)	20.1 (17.1–23.4)	21.2 (17.3–25.7)	21.3 (17.9–25.1)	17.5 (13.3–22.6)
South	44.4 (40.7–48.3)	42.8 (38.9–46.8)	44.0 (39.9–48.3)	39.8 (36.4–43.3)	40.0 (31.3–49.4)
West	12.8 (11.1–14.9)	13.2 (11.0–15.8)	12.4 (10.6–14.5)	15.2 (12.8–17.9)	18.9 (11.4–29.7)
**Insurance status^b^**					
Medicare	44.2 (41.4–47.1)	42.8 (40.5–45.1)	44.7 (42.3–47.1)	42.9 (40.8–45.0)	44.1 (41.2–46.9)
Medicaid	7.9 (6.7–9.3)	7.3 (6.2–8.6)	8.8 (7.5–10.3)	9.9 (8.6–11.3)	12.2 (10.4–14.2)
Private insurance	37.2 (34.5–39.9)	39.0 (36.2–41.9)	36.0 (34.0–38.0)	35.2 (33.1–37.3)	31.1 (28.8–33.5)
All other	10.7 (9.2–12.4)	10.9 (9.2–12.9)	10.6 (9.1–12.3)	12.1 (10.7–13.6)	12.6 (10.9–14.6)

a Totals here are less than totals for the other characteristics due to missing values; 98,887 (15.1%) in 1995–1997; 126,115 (16.4%) in 1998–2000; 184,035 (19.8%) in 2001–2003; 205,428 (21.8%) in 2004–2006; and 258,436 (17.7%) in 2007–2010.

b Totals here are less than totals for the other characteristics due to missing values; 13,617 (2.1%) in 1995–1997; 14,766 (1.9%) in 1998–2000; 14,707 (1.6%) in 2001–2003; 21,493 (2.3%) in 2004–2006; and 24,277 (1.7%) in 2007–2010.

Black women in all 3 age groups and across all 5 periods had significantly higher crude rates of hospitalizations for hypertension than did white women (Table 2). Among women aged 18 to 44 in 2007–2010, whites had a hospitalization rate of 24.1 per 100,000 population, and blacks, 128.1 per 100,000 population. The rates of these hospitalizations increased with age. For almost every subgroup, rates were flat across the 5 periods; we found a positive linear trend only for white women aged 65 or older. Black men overall had significantly higher crude rates of hospitalizations for hypertension than did white men, a pattern similar to that seen in women (Table 3). The rates of these hospitalizations increased with age, and in all age–race categories, the rates did not differ significantly across the 5 periods. 

**Table 2:** Crude Preventable Hospitalization Rates for Hypertension Per 100,000 Population Among Women, by Age and Race, National Hospital Discharge Survey, United States, 1995–2010^a^

Age/Race	Period, Rate (95% Confidence Interval)				

	1995–1997	1998–2000	2001–2003	2004–2006	2007–2010
**18–44 y**					
White	16.0 (11.5–20.5)	17.3 (13.0–21.6)	14.6 (10.1–19.2)	17.2 (12.0–22.4)	24.1 (13.6–34.5)
Black	87.5 (60.3–114.7)	111.3 (85.6–137.0)	101.5 (78.5–124.5)	104.4 (78.9–130.0)	128.1 (90.6–165.6)
**45–64 y**					
White	107.3 (91.3–123.4)	121.7 (100.6–142.9)	127.2 (105.2–149.2)	114.3 (96.9–131.7)	99.3 (80.7–118.0)
Black	494.2 (406.0–582.3)	401.3 (314.7–487.8)	486.6 (381.0–592.2)	475.5 (385.5–565.6)	553.0 (399.0–707.0)
**≥65 y**					
White^b^	248.9 (211.9–286.0)	266.6 (231.4–301.7)	333.7 (275.9–391.5)	311.3 (267.3–355.2)	361.2 (277.5–444.9)
Black	595.5 (421.3–769.7)	897.7 (668.3–1127.1)	798.3 (621.0–975.6)	640.4 (507.1–773.7)	779.6 (589.3–969.9)

a Difference between whites and blacks is significant (*P* < .05) for all categories and all periods. Calculated by using *z* test.

b Increasing linear trend is significant (β = +26 per 100,000 population per period; *P* = .03).

**Table 3:** Crude Preventable Hospitalizations Rates for Hypertension Per 100,000 Population Among Men, by Age and Race, National Hospital Discharge Survey, United States, 1995–2010^a^

Age/Race	Period, Rate (95% Confidence Interval)				

	1995–1997	1998–2000	2001–2003	2004–2006	2007–2010
**18–44 y**					
White	20.9 (16.8–25.0)	20.7 (15.5–25.8)	26.0 (19.2–32.9)	23.2 (18.1–28.4)	21.0 (12.4–29.7)
Black	73.2 (52.5–93.9)	93.5 (70.3–116.7)	98.7 (71.5–125.9)	106.8 (78.3–135.3)	88.4 (64.3–112.4)
**45–64 y**					
White	88.6 (73.5–103.6)	104.8 (87.9–121.6)	104.1 (88.4–119.9)	100.3 (82.1–118.6)	125.4 (92.6–158.3)
Black	357.1 (287.5–426.7)	393.4 (313.0–473.9)	387.6 (283.5–491.7)	351.2 (273.5–429.0)	465.0 (349.6–580.5)
**≥65 y**					
White	184.0 (150.3–217.7)	149.2 (116.4–182.1)	186.5 (146.5–226.5)	173.1 (140.7–205.5)	233.5 (177.6–289.5)
Black	364.8 (236.7–493.0)	500.5 (288.3–712.8)	692.6 (424.0–961.2)	377.7 (243.5–511.9)	675.1 (461.4–888.8)

a Difference between whites and blacks is significant (*P* < .05) for all categories and all periods. Calculated by using *z* test.

Age- and sex-standardized rates confirmed that blacks had higher rates than whites (Figure). For example, in 2007–2010, whites had a rate of 97.4 per 100,000 population; blacks had a rate of 334.2 per 100,000 population — more than 3 times the rate for whites. Neither racial group showed a significant linear trend over the 5 periods.

**Figure:**
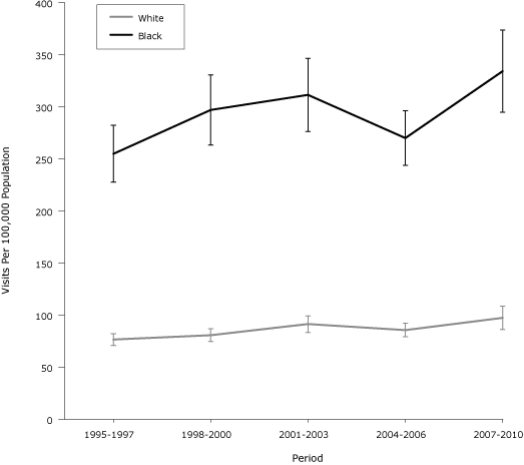
Age- and sex-standardized preventable hospitalization rates for hypertension among adults in the United States, by race, National Hospital Discharge Survey, 1995–2010. Difference between whites and blacks was significant for all periods (calculated by using a *z* test). Error bars indicate confidence intervals. **Table:** 

Race	Year, n (95% Confidence Interval)				

	1995–1997	1998–2000	2001–2003	2004–2006	2007–2010
White	76.7 (70.9–82.3)	80.76 (74.7–86.9)	91.4 (83.5–99.3)	85.7 (79.2–92.2)	97.4 (86.1–108.7)
Black	254.9 (227.6–282.2)	296.9 (263.3–330.5)	311.4 (276.2–346.6)	269.9 (243.8–296.0)	334.2 (294.7–373.7)

## Discussion

We examined recent national data and found that blacks in every age and sex subgroup had higher rates of preventable hospitalizations for hypertension than did whites. This finding was confirmed across all 5 study periods; age- and sex-standardized rates were, at a minimum, 3 times as high for blacks as for whites. Given the higher prevalence of hypertension among blacks than whites, higher hospitalization rates may be expected. However, the prevalence for hypertension for blacks aged 18 or older in 2005–2008 was 38.6% and 32.3% for whites (21), so it appears that blacks may have proportionately higher rates of hospitalizations than whites.

Other investigators have reported racial differences in hospitalizations for hypertension but none in a sample that represented hospitalizations from all 50 states. An analysis of hospital data from 1991–1998 in California for people aged 20 to 64 indicated that unadjusted rates for non-Hispanic black men were 7 times higher than rates for non-Hispanic white men; rates for non-Hispanic black women were 8 times higher than rates for their non-Hispanic white counterparts (10). An analysis of 1997 data from a similar-aged population of non-Hispanics from 22 states indicated that black men were 6.5 times as likely to be hospitalized for hypertension as white men, and black women were 8 times as likely to be hospitalized as white women (12). In 2003, hospitalizations for people aged 18 or older from 23 states were analyzed; after adjustments for age and sex, non-Hispanic black men and women combined were 5 times as likely to be hospitalized for hypertension as non-Hispanic white men and women combined (13). Similar results have been found for people aged 65 or older by using Medicare data from Maryland in 2006; blacks were 3 times as likely to be hospitalized for hypertension as whites (11). In addition to disparities in hypertension prevalence, the studies just described propose a number of possible explanatory factors, including poor access to care due to lack of health insurance; various socioeconomic (eg, low income) and geographic barriers; inadequate care, including medication issues and lifestyle factors; and more serious disease, including the presence of comorbid conditions (10–12). The outpatient care system and the public health system may need to be strengthened simultaneously to narrow these racial differences in rates. A recent editorial suggested that hospitals should not be punished for high rates of preventable hospital and emergency department visits; instead, a comprehensive community approach to improve access will most likely be effective in lowering the number of visits (22).

Although earlier studies showed racial differences in hospitalizations for hypertension, this study contributes to the literature in the following ways: 1) the data are from hospitals representative of the 50 states and the District of Columbia, 2) age- and sex-specific rates are provided for multiple periods, establishing a baseline for future monitoring of health disparities, and 3) confidence intervals are provided, showing the degree of certainty of point estimates and allowing judgments about the significance of differences between subpopulations.

This study has limitations. First, race data were missing for 15% to 22% of hospitalizations, so we did not include these hospitalizations in our analysis. Depending on the distribution of the hospitalizations that had missing race data, their exclusion could have led to higher or lower differences in rates between blacks and whites; however, we believe that it is unlikely that the distribution would be so skewed as to eliminate the differences between the 2 groups (19). For example, using the period 2004–2006 and assigning all missing hospitalizations to whites, we observed an increase in the rate for whites from 91 per 100,000 population to 129 per 100,000 population. Even after such an extreme skewing of the distribution, the rate for blacks was still almost 2 times as high (252 per 100,000 population) as the rate for whites. Second, starting in 2000, the NCHS allowed abstractors to record multiple races in the database; people classified before 2000 as either black or white were likely classified as multiracial starting in 2000. Because less than 1% of the records we used classified race as multiracial, the effect of excluding them from the analysis could only have been minimal. The difference between blacks and whites was smaller in our study than in previous studies, most likely because we did not exclude Hispanics from the categories of blacks and whites (other studies have compared non-Hispanic whites and non-Hispanic blacks). Also, as required by AHRQ’s specifications, all hypertension hospitalizations that occurred because of transfers from another facility (hospital, skilled nursing facility, or another health facility) for the years 1995 through 2000 were not excluded because transfer data were not collected during those years. However, on the basis of available transfer data (2001 through 2010) on hypertension hospitalizations and because transfer percentages ranged by year from 1.9% to 3.0%, we believe that only a small error was created. Finally, the use of half-samples by NCHS in 2008, 2009, and 2010 may have created some instability in the trends. Without additional time points, assessing the effect that this has had on our results is difficult.

This research establishes a solid baseline, allowing for ongoing monitoring of preventable hospitalizations for hypertension, an ACSC that may be reduced as the PPACA of 2010 is implemented. Many of these reforms are aimed at increasing access to care for all racial groups, expanding primary prevention and early diagnosis of hypertension, and improving hypertension treatment. These types of interventions are believed by many clinical experts to reduce the use of hospitals for dangerously uncontrolled hypertension (7,23,24). If these efforts are successful, we suspect that hypertension hospitalization rates will decrease and the gap between the rates for blacks and whites will be reduced. Future research, which will require socioeconomic and other data from multiple sources and the use of multivariate analyses, should go beyond surveillance and focus on explanatory models for these disparities.
